# Ellipsometric Surface Oxidation Model of ALD-Grown Vanadium Oxide Mixed-Valence System

**DOI:** 10.3390/nano15090645

**Published:** 2025-04-24

**Authors:** Xiaojie Sun, Shuguang Wang, Qingyuan Cai, Jingze Liu, Changhai Li, Ertao Hu, Jing Li, Songyou Wang, Yuxiang Zheng, Liangyao Chen, Youngpak Lee

**Affiliations:** 1 State Key Laboratory of Photovoltaic Science and Technology, Department of Optical Science and Engineering, School of Information Science and Technology, Fudan University, Shanghai 200433, Chinayplee@hanyang.ac.kr (Y.L.); 2 Shanghai Key Laboratory of Optical Coatings and Spectral Modulation, Shanghai Institute of Technical Physics, Chinese Academy of Sciences, Shanghai 200083, China; 3 College of Electronic and Optical Engineering, Nanjing University of Posts and Telecommunications, Nanjing 210023, China; 4 High Tech Center for New Materials, Novel Devices and Cutting-Edge Manufacturing, Yiwu Research Institute, Fudan University, Yiwu 322000, China; 5 Department of Physics, Quantum Photonic Science Research Center, Hanyang University, Seoul 04763, Republic of Korea

**Keywords:** vanadium oxides, mixed-valence system, surface oxidation, optical thin film, atomic layer deposition, spectroscopic ellipsometry, optical property

## Abstract

Vanadium and oxygen form a complex system of vanadium oxides with multiple phases and mixed valency, increasing the difficulty of characterization. In this work, amorphous vanadium oxide thin films with mixed valence states were fabricated by atomic layer deposition, and then post-annealing was conducted for crystalline films. For the surface analysis of this mixed-valence system, X-ray photoelectron spectroscopy (XPS) and Auger electron spectroscopy (AES) were employed. However, XPS is only able to quasi-quantitatively determine the surface-proximity oxidation states. To account for the inadequacy of surface-sensitive XPS and AES techniques, a surface oxidation model (SOM) was proposed for the ellipsometric modeling of the mixed-valence system. Furthermore, by conducting air thermal oxidation (ATO) experiments, the four sets of fitting parameters of SOM were decreased to three, lowering the system complexity. This study is expected to help with the analysis of vanadium oxide mixed-valence systems and other multivalent metal oxide systems.

## 1. Introduction

Vanadium is a multivalent transition metal that forms a large number of vanadium-based compounds, including vanadium oxides, vanadates, vanadium halides and so on [1,2]. Vanadium oxides, particularly vanadium dioxide (VO_2_), vanadium trioxide (V_2_O_3_) and vanadium pentoxide (V_2_O_5_), have attracted much attention because of their wide applications. In the vanadium–oxygen system, vanadium and oxygen atoms can compound into vanadium oxides of various chemical states, including valence states and crystal structures. For example, the well-known vanadium dioxide [3,4,5] is a phase change material (PCM) that can demonstrate different properties between two phases, which results from the change of crystal structure because of external stimuli. The most common phase of VO_2_ is called the monoclinic (M1) phase belonging to the space group *P2_1_/c*, where VO_2_ (M1) behaves like a typical semiconductor, with minor absorption in the optical wavelengths. When the ambient temperature rises above 68 °C, the crystal structure of VO_2_ changes to a rutile-like tetragonal phase with a space group of *P4_2_/mnm*, which is denoted as the R phase. During this phase change, the physical properties of VO_2_ demonstrate huge variations, such as its electrical conductivity and optical constants. From a macroscopic viewpoint, VO_2_ (R) behaves like a metal, which is known as the metal-insulator transition (MIT) of VO_2_ [6,7].

Except from these two most common phases, at least 12 other phases of VO_2_ have been proposed [1]. As for other valence states, each vanadium oxide has its own multiple polymorphs, which makes the vanadium–oxygen system more complicated. It is reported that there are in total 52 stable and meta-stable phases in the vanadium–oxygen system [1]. Researchers have been dedicating themselves to controlling the stoichiometry of vanadium oxides [8,9,10,11] to ensure the quality of fabricated samples and their further applications. For example, VO_2_ has been used for smart windows [12,13,14,15,16] and smart radiative coolers [17,18,19,20,21] because of its phase-change ability [22,23,24,25,26]. However, VO_2_ is easily oxidated into V_2_O_5_ in the air [27,28], which will undermine the effectiveness of fabricated devices and might cause degradation with time. Therefore, precise control of the stoichiometry of VO_2_ is of vital importance. Furthermore, if natural aging is unavoidable, precise determination of the separate properties of VO_2_ and V_2_O_5_ will be useful for both design and fabrication of VO_2_-based thermochromic devices. In this work, vanadium oxide thin films were grown by atomic layer deposition (ALD), with crystalline phases characterized by X-ray diffraction (XRD) and Raman scattering spectroscopy. The surface properties of the mixed-valence system were analyzed by X-ray photoelectron spectroscopy (XPS) and Auger electron spectroscopy (AES). To account for the inadequacy of the XPS technique, a surface oxidation model (SOM) was proposed to fully represent the vanadium oxide thin films with a surface oxidation layer and a surface roughness layer. SOM was used in the inverse fitting of the spectroscopic ellipsometry (SE) data, and different dispersion models were employed to extract the temperature-dependent optical properties of these vanadium oxide thin films. To increase the credibility of the SOM, air thermal oxidation (ATO) experiments were conducted as a supplement. This study will help with the analysis of the vanadium oxide mixed-valence system and other multivalent metal oxide systems. We believe that the synthesis of the vanadium–oxygen family and their future applications will all benefit from a clear understanding of their optical properties.

## 2. Materials and Methods

### 2.1. Thin Film Fabrication

**Figure 1:**
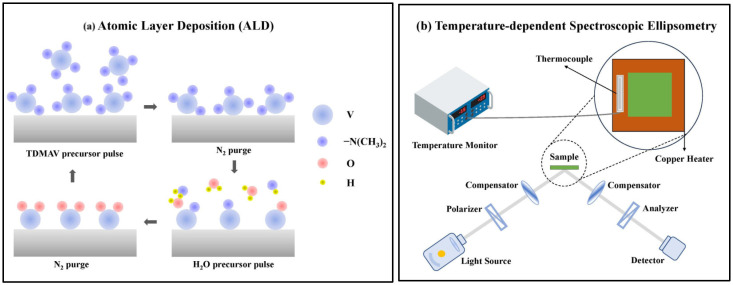
(**a**) Schematic of the ALD. (**b**) Schematic of the temperature-dependent spectroscopic ellipsometry.

Vanadium oxide thin films of different thicknesses were prepared by ALD (BENEQ, TFS500, Espoo, Finland) [29,30,31,32,33,34]. The schematic of ALD is depicted in Figure 1a. The precursor of vanadium was chosen to be tetrakis-dimethyl-amino vanadium (TDMAV) purchased from Infinity Scientific (London, UK). The precursor of oxygen was chosen to be H_2_O, and the purge gas was N_2_. Films were deposited on silicon and silica substrates. After trial experimentation, the temperature of the TDMAV source was kept at 60 °C to avoid precursor degradation, and the reactor temperature was chosen to be 200 °C, for higher deposition temperature may produce a CVD-type (chemical vapor deposition) growth [32]. The flow rates of N_2_ carrier gas and purge gas were 300 and 200 sccm (standard cubic centimeters per minute), respectively. For our ALD reactor, the time sequence of t1–t2–t3–t4 was determined to be 1s–3s–0.5s–3s, where t1 and t3 are the pulse time of TDMAV and H_2_O, t2 and t4 are the purge time of N_2_ gas after each precursor exposure. One full execution of a time sequence is called a cycle, and deposition cycles from 300 to 1500 were experimented to determine the growth rate per cycle (GPC), as we will show later in the Results and Discussion section.

The ALD-grown films were post-annealed in a tube furnace (BEQ, BTF-1200C-SL, Hefei, China). The annealing temperature and time were 475 °C and 90 min. The vacuum pressure was kept at 0.1 Pa to ensure an oxygen-deficient condition [10].

The samples of ATO experiments were fabricated by heating in the atmosphere using a hot plate. To ensure a total conversion into the highest-oxidation-state V_2_O_5_, the heating temperature and time were set as 400 °C and 4 h, respectively [28]. All samples in this work are listed and numbered in Table 1.

### 2.2. Thin Film Characterization

Temperature-dependent spectroscopic ellipsometry was conducted on the V-VASE instrument (J. A. Woollam Co., Lincoln, NA, USA). All samples were measured within the temperature range of 30~120 °C. The photon energy range was 0.5–3.0 eV, with a step of 0.05 eV. Three incident angles, 65°, 70° and 75°, were adopted. A self-built thermal heating system was connected to the ellipsometer sample stage for temperature control. A schematic of the temperature-dependent spectroscopic ellipsometry is shown in Figure 1b. Surface and cross-sectional scanning electron microscopy (SEM) was conducted for surface morphology and determining the film thickness, using a GeminiSEM 300 instrument (Zeiss, Oberkochen, Germany). Atomic force microscopy (AFM) was conducted on a Dimension Icon (Bruker, Bremen, Germany). To determine the mixed valence states of these vanadium oxide thin films, XPS and AES were measured on a K-Alpha instrument (Thermo Scientific, Waltham, MA, USA) using an Al Kα monochromatic source (1486.6 eV). Raman spectroscopy (LabRAM, Horiba, Kyoto, Japan) was used to characterize the phases of the fabricated samples by comparing them with the standard characteristic peaks [1,35,36]. For the crystalline structures, we conducted XRD on a SmartLab SE (Rigaku, Tokyo, Japan).

### 2.3. Methods

The as-deposited samples should have a valence of around 4, because this is the valence state of the precursor TDMAV, with consideration of oxidizing processes during the ALD deposition, like high temperature and oxygen precursor. Furthermore, V (4+) is not the highest valence state of the vanadium–oxygen system, so these samples will undergo slow oxidation into V (5+) in the ambient air atmosphere. Therefore, we propose that the obtained vanadium oxide samples are a mixture of two valence states, where the surfaces are of higher valence than the interior of the sample films owing to air exposure. To determine the specific ratio of these two valences, XPS, as a well-known surface analysis tool with the ability to determine different chemical states, was used to analyze the surface-proximate properties of the vanadium oxide thin films [37,38,39]. However, XPS is known to be mostly sensitive to the first ~10 nm below the film surface and also suffers from inaccurate quantitative determination [27,28]. Owing to its low probing depth and surface sensitivity, we only use the XPS technique for a semi-quantitative analysis, and instead propose a surface oxidation model (SOM) for a more accurate determination of the mixed-valence system. In this model, the vanadium oxide thin films are adequately represented as a multi-layered film structure, with a surface oxidation layer and a surface roughness layer on top, a VO_2_ layer in between, and the substrate layers underneath. As detailed in the previous section, the optical properties of the vanadium oxide thin films were characterized by temperature-varying SE. The proposed SOM was used in the inverse optimization fitting of the ellipsometric data, and different dispersion models were resorted to for different temperatures to ensure a temperature-different analysis. There were four sets of independent fitting parameters, that is, the thickness of the surface roughness layer, the optical constants of V_2_O_5_, the optical constants of VO_2_, and their relative thickness ratio. To increase the trustworthiness of the optimization based on SOM, we lowered the fitting parameters to just three by performing further experiments called air thermal oxidation (ATO), in which vanadium oxide thin films were thermally oxidized in the air into a full V (5+) valence state. Separate and independent ellipsometric characterizations were performed to determine the optical constants of these fully oxidized V_2_O_5_ samples.

## 3. Results and Discussion

### 3.1. Film Deposition and Post-Annealing

**Figure 2:**
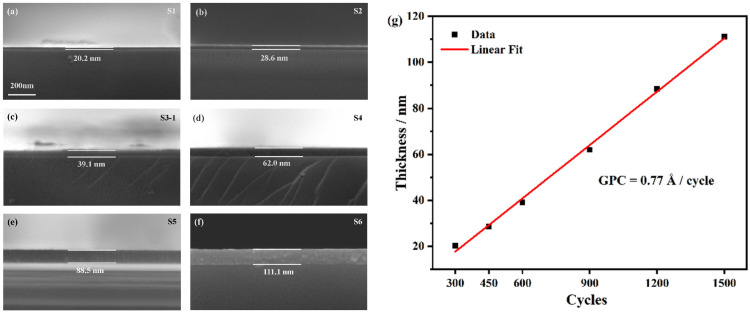
(**a**–**f**) Cross-sectional SEM images of samples S1–S6 with deposition cycles of 300, 450, 600, 900, 1200 and 1500, respectively. Their thicknesses were extracted by comparing them to the scale bar. (**g**) The thickness data versus the deposition cycles, where black dots are experiment data, and the red line is the linear fit of them. The slope of this linear fit is the GPC of the ALD deposition, as denoted in the figure.

Six vanadium oxide thin film samples with different thicknesses and cycles were fabricated by ALD: namely, 300, 450, 600, 900, 1200 and 1500 cycles. They are labeled as S1–S6. In Figure 2a–f, the cross-sectional SEM pictures are shown for these samples, with their thicknesses measured by comparing them to the scale bar. Surface and cross-sectional SEMs testify that the uniformity of the surface morphology and cross-sectional distribution is rather good. By using the thickness data of samples S1–S6 extracted from the cross-sectional SEM, we can determine the GPC, which is an essential parameter for ALD thin film deposition. The GPC is shown in Figure 2g, with the R-square of the linear fitting as 0.997, which implies a relatively good ALD growth up to hundreds of nanometers.

**Figure 3:**
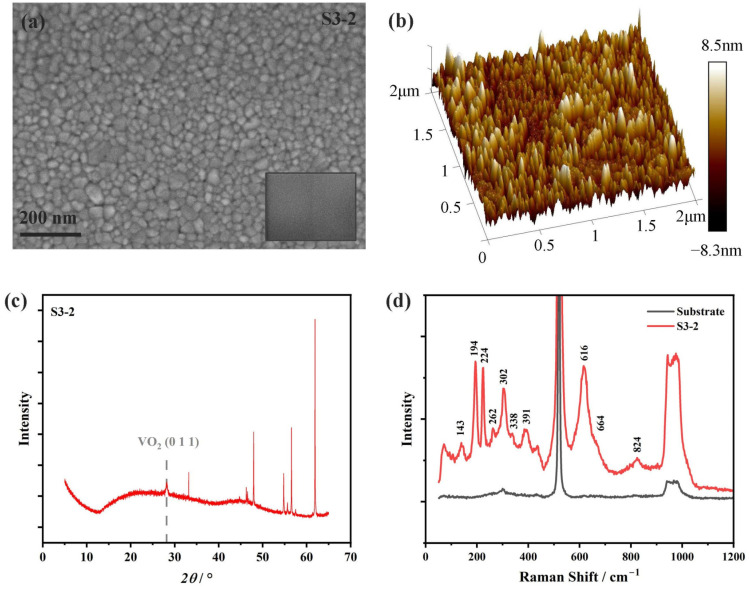
(**a**) Surface SEM of the post-annealed film sample S3-2, where crystallites can be clearly seen. The inset shows the film surface morphology before annealing for comparison. (**b**) AFM measurement of the post-annealed film sample S3-2, with a measured surface roughness of 1.76 nm. (**c**) XRD of the post-annealed sample S3-2 with an annealing temperature of 475 °C. The diffraction peak at 28.2 ° is from the (0 1 1) orientation of VO_2_ (M1). (**d**) Raman spectra of the annealed vanadium oxide sample S3-2 (red line) and the silicon substrate (black line). Characteristic Raman scattering peaks of VO_2_ (M1) are annotated in the figure. The strongest peak at about 500 cm^−1^ and the flattened peak at about 1000 cm^−1^ are from the silicon substrate.

The films obtained by direct ALD growth were of an amorphous nature. To increase the crystallinity and form the preferred orientation, post-deposition thermal annealing was conducted through a tube furnace, with an annealing temperature of 475 °C and a time of 90 min. The annealing was conducted in a vacuum, with a pressure of 0.1 Pa. In this oxygen-deficient condition, the film samples cannot absorb oxygen from the ambient, so post-annealing was able to lower the oxidation state of the vanadium oxide films. The film sample of 600 deposition cycles and a thickness of ~40 nm was post-annealed and labeled as S3-2, and its surface SEM picture is shown in Figure 3a, with both small-scale and large-scale uniformity. We also performed the AFM measurement, as can be seen in Figure 3b, with a measured surface roughness of 1.76 nm. According to the XRD measurements, films annealed at 475 °C demonstrated good crystallinity, as can be seen from Figure 3c. By comparing with PDF card no. 19-1401, we know that the diffraction peak at 28.2° is the preferred orientation (0 1 1) of VO_2_ (M1). The Raman spectra are shown in Figure 3d, with all 10 distinguishable characteristic scattering peaks belonging to the M1 phase of VO_2_ [1,35]. Both XRD and Raman spectroscopy characterizations indicate that the vanadium oxide thin films are of a single valence state and crystal structure. However, these two techniques were unsuitable for trace analysis, so XPS, as a well-known surface analysis technique [40], was used for a more accurate characterization.

### 3.2. XPS Characterization and Ellipsometry Modeling

**Figure 4:**
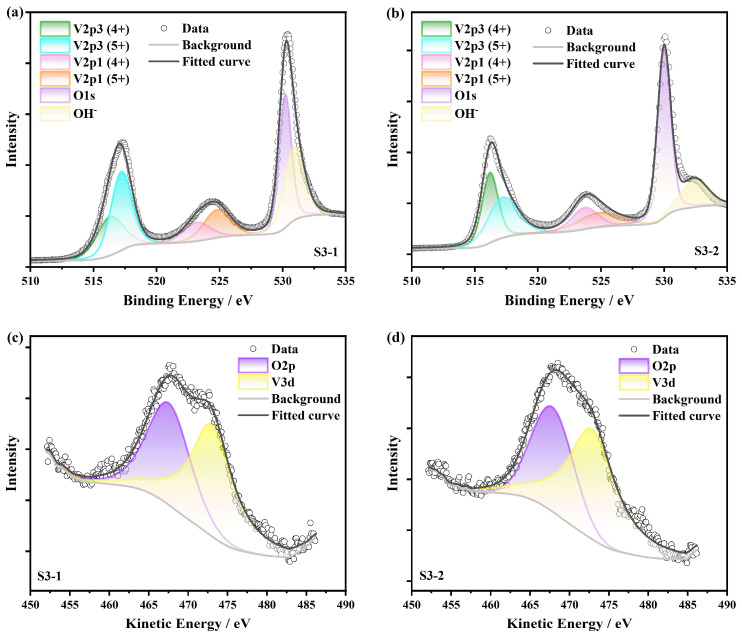
(**a**,**b**) XPS spectra of the vanadium oxide films before and after annealing (S3-1 and S3-2), respectively. Dots represent the experiment data. The dark grey line is the fitted curve, and the light grey line is the background. (**c**,**d**) Auger spectra of the vanadium oxide films before and after annealing (S3-1 and S3-2), respectively.

XPS was conducted for samples S3-1 and S3-2. The narrow scans of the XPS spectra before and after annealing and their deconvolutions are depicted in Figure 4a,b. In the binding energy range from 510 to 535 eV, two energy levels, V2p and O1s, can be found. Owing to spin-orbital splitting (SOS), the V2p energy level is further split into two correlated peaks: namely, V2p_3/2_ and V2p_1/2_. In the XPS spectra fitting of vanadium oxides, the O1s energy level at 530 eV has been shown to be a better reference than the C1s level of adventitious carbon [27,28]. In the O1s envelope located near 530 eV, two peaks can be deconvoluted, of which the first one (530 eV) is the oxygen in metal oxides, and the second one with a higher binding energy (530.5~532 eV) can be ascribed to surface organics contamination [28]. The area ratio of the V2p_3/2_ and V2p_1/2_ energy levels is set as 2:1 with consideration of their physical origin. For different oxidation states of vanadium, their binding energies show a clear distribution [27], which is why XPS can be utilized in chemical state analysis. By comparing the V2p_3/2_ XPS spectra of samples S3-1 and S3-2, one can clearly see that the ratio between V4+ and V5+ components changed. During the fitting, the binding energies of V2p_3/2_ and V2p_1/2_ of V5+ are chosen to be 517.2 and 524.8 eV, and those of V4+ are 516.3 and 523.5 eV, respectively [27]. We also analyzed the LMM Auger peak in the XPS spectra, which originates from the O2p and V3d Auger electrons [41], as shown in Figure 4c,d. The area ratio of V5+ and V4+ XPS peaks, and the area ratio of O2p and V3d Auger peaks, can be used as a semi-quantitative way of determining the mixed valence states within the scope of the XPS detection range, as tabulated in Table 2.

**Figure 5:**
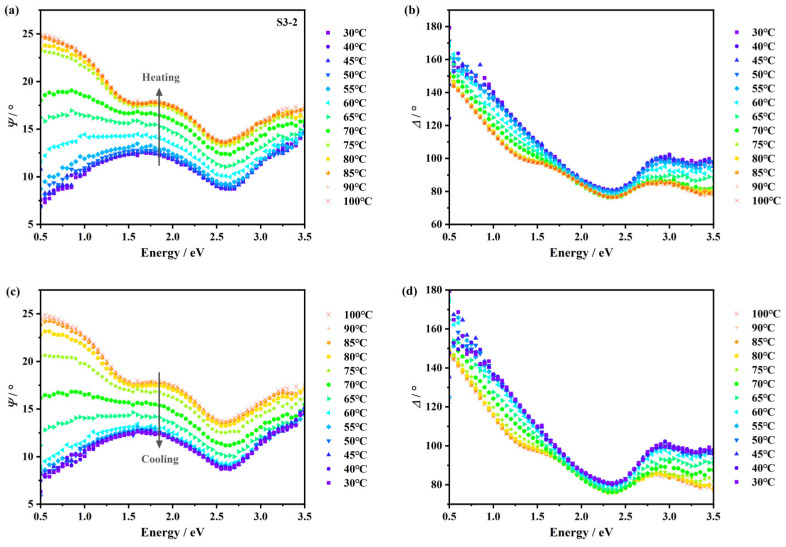
The ellipsometric data of sample S3-2 under the incident angle of 70°. (**a**) The Ψ data during the heating process. (**b**) The Δ data during the heating process. (**c**) The Ψ data during the cooling process. (**d**) The Δ data during the cooling process.

SE is a characterization technique that can determine both the optical properties and thickness of the thin film [42,43,44,45,46]. Unlike XPS, SE is not a surface-sensitive-only technique but can be used to analyze the interface properties of multi-layer thin films. Detailed information about ellipsometric angles Ψ and Δ, Drude and Tauc–Lorentz models, is provided in Appendix A. The temperature-dependent Ψ and Δ ellipsometric data of sample S3-2 under the incident angle of 70° are shown in Figure 5, where the heating process is depicted in (a) and (b), and (c) and (d) are for the cooling process. A gradual shift and a thermal hysteresis behavior can be observed. In actual measurements, ellipsometry was performed under three incident angles, so as to increase data redundancy and fitting accuracy. By combining Equations (A1) and (A2) with Fresnel equations, with an inverse fitting optimization procedure, the optical properties and thickness of the thin film can be extracted. For spectroscopic optical properties, dispersion models are needed to decrease the fitting parameters. Here, for vanadium oxides at low temperatures, we used the Tauc–Lorentz model to mimic its semiconductor-like response, and the Drude–Tauc–Lorentz model was used for high-temperature vanadium oxides for its metal-like response [47,48,49,50].

### 3.3. Surface Oxidation Model and Air Thermal Oxidation

**Figure 6:**
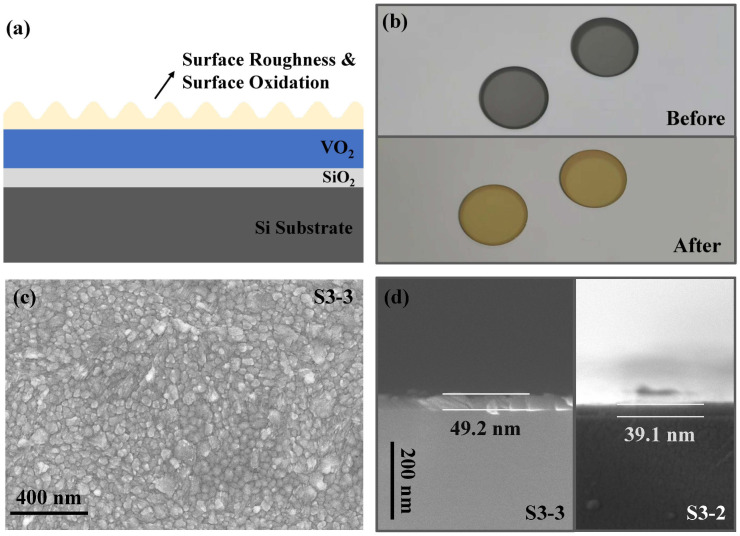
(**a**) Schematic of the SOM. (**b**) Vanadium oxide film deposited on silica substrate before and after ATO. (**c**) Surface SEM image of the film deposited on Si after ATO (S3-3). (**d**) Cross-sectional SEM image of the film deposited on Si after (S3-3) and before (S3-2) ATO.

SE is an indirect characterization technique owing to its model-dependent nature [45]. The film parameters, like optical constants and thickness, have to be extracted from inverse fitting optimizations. Due to this indirectness, the physical model constructed in the optimization to represent the film structure should be based on an accurate understanding of the surface and interface properties. In this study, we conducted both XPS and SE characterizations, for the directness of XPS and the quantitative nature of SE can complement each other. To be more specific, from the XPS characterization, we know that the vanadium oxide films consist of two valence states, V4+ and V5+, and the surface has a higher valence than the interior because of air oxidation. On the basis of this observation, we construct a surface oxidation model (SOM) for ellipsometry optimization, in which the vanadium oxide films are divided into a surface oxidation layer of V_2_O_5_, a surface roughness layer and an interior layer of VO_2_. There are four independent fitting parameters; that is, the thickness of the surface roughness layer, the optical constants of V_2_O_5_, the optical constants of VO_2_, and their relative thickness ratio. We reduced this four-parameter model to a three-parameter model by further conducting air thermal oxidation (ATO) experiments, in which the mixed-valence vanadium oxide samples were fully converted into V_2_O_5_ by thorough heating in the air (labeled as S3-3). In this way, a mixed-valence system was oxidated into a low-complexity single-valence system. By predetermining the optical constants of V_2_O_5_ of this single-valence system, the fitting parameters of the SOM can be decreased to three. The schematic of SOM is depicted in Figure 6a, where the vanadium oxide films have a surface oxidation and roughness layer of V_2_O_5_ on top, and the surface oxidation of the Si substrate was also considered. In Figure 6b, the vanadium oxide film deposited on the silica substrate before and after ATO is shown, where a yellow color of pure V_2_O_5_ can be observed [2]. In Figure 6c,d, the surface and cross-sectional SEM of the film deposited on Si after ATO are shown. We can observe that the film formed a polycrystalline structure, and its thickness increased by about ten nanometers after ATO because of oxygen absorption.

**Figure 7:**
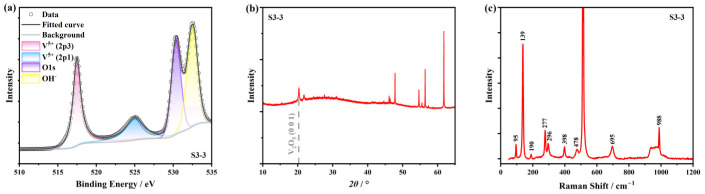
(**a**) XPS spectrum of the ATO sample S3-3. (**b**) XRD result of the ATO sample S3-3. A characteristic diffraction peak at 20° belongs to the (0 1 1) orientation of α-V_2_O_5_. (**c**) Raman scattering spectrum of the ATO sample S3-3. Characteristic peak positions of α-V_2_O_5_ are annotated in the figure. The strongest peak at about 500 cm^−1^ and the flattened peak at about 1000 cm^−1^ are from the Si substrate.

**Figure 8:**
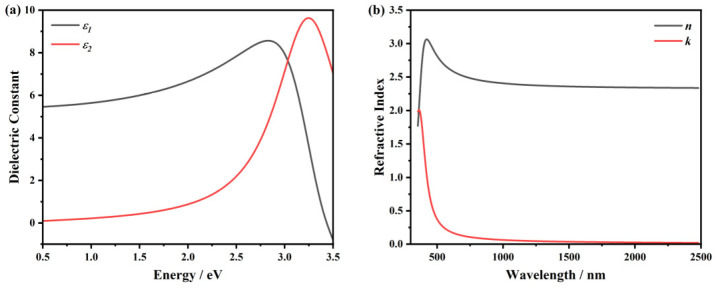
Optical properties of sample S3-3 and the surface V_2_O_5_ layer in the SOM. (**a**) Dielectric constants, where the black line refers to the real part, and the red line refers to the imaginary part. (**b**) Complex refractive index, where the black line is the refractive index, and the red line is the extinction coefficient.

**Figure 9:**
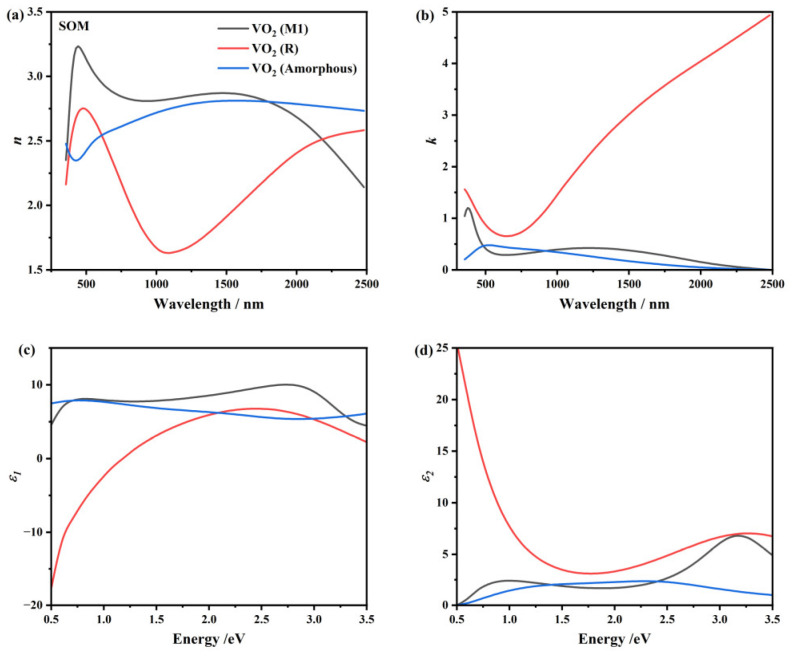
Optical properties of the interior VO_2_ layer in the SOM. (**a,b**) Refractive index and extinction coefficient, respectively. (**c,d**) Real and imaginative parts of the dielectric constants, respectively. Black, red and blue lines are for VO_2_ (M1, S3-2), VO_2_ (R, S3-2) and VO_2_ (Amorphous, S3-1), respectively.

We also conducted other characterizations on the ATO samples. In Figure 7a, the XPS spectrum of S3-3 is depicted, where a total conversion into V5+ can be observed. In Figure 7b, the XRD result of S3-3 is shown, where the preferred orientation of V_2_O_5_ (0 0 1) at 20° is the most prominent diffraction peak of the film [51]. Figure 7c presents the Raman scattering spectrum of S3-3, in which all the characteristic peaks can be ascribed to the α phase of V_2_O_5_ (α-V_2_O_5_) [1,36], confirming the thorough oxidation of the ATO samples. After these characterizations, SE was conducted on sample S3-3 to obtain the ellipsometric data, and the optical properties of V_2_O_5_ are shown in Figure 8. Detailed fitting parameters of sample S3-3 consisted of V_2_O_5_ are listed in Table A1. The Tauc–Lorentz model was used to extract the optical constants of V_2_O_5_ [51,52], which was then applied in the SOM as the optical constants of the surface oxidation and roughness layer. Then, the thickness of the surface roughness layer and the thickness and optical properties of the interior VO_2_ layer before (S3-1) and after (S3-2) annealing are fitted in the inverse optimization, which is shown in Table 3 and Figure 9, respectively. We can observe that the low-temperature phases, that is, the amorphous and M1 phases, do not differ much in their optical properties, except that the M1 phase has more absorption in the high-energy band (see Figure 9b). Correspondingly, in the wavelength scale, the absorption is in the ultra-violet band. As for the high-temperature R phase, we can observe a typical semi-metal response. Detailed fitting parameters of sample S3-1 and S3-2 are listed in Table A1. 

## 4. Conclusions

In this work, vanadium oxide thin films with mixed valence states were fabricated by ALD. Multiple characterization techniques were used for a comprehensive analysis of the mixed-valence system, including SEM, AFM, XRD, Raman scattering spectroscopy, XPS, AES and temperature-dependent SE. XPS and AES were conducted to analyze the mixed valence states near the sample surface; however, the film interior was out of their detection range. To account for the inadequacy of surface-sensitive XPS and AES techniques, a surface oxidation model (SOM) was proposed for the ellipsometric modeling of the mixed-valence system. Furthermore, by conducting air thermal oxidation (ATO) experiments, the fitting parameters of SOM were decreased to two, lowering the system complexity. In the end, the separate thickness and optical properties of vanadium dioxide and vanadium pentoxide were determined based on SOM and ATO. This study is expected to help with the analysis of vanadium oxide mixed-valence systems and other multivalent metal oxide systems.

## Figures and Tables

**Table 1:** Samples deposited, processed and analyzed throughout this work. Firstly, a set of amorphous (“Amo”) samples with increasing thickness were deposited by ALD, and they were labeled as S1–S6, respectively. Then, sample S3-1 (deposition cycles of 600 and estimated thickness of 40 nm) was post-annealed (“Ann”) to increase crystallinity and labeled as S3-2. Furthermore, in the Air Thermal Oxidation (ATO) experiments, sample S3-2 was further oxidized into the highest valence state, and was labeled as S3-3.

ALD Cycles	300 Cycles	450 Cycles	600 Cycles	900 Cycles	1200 Cycles	1500 Cycles
**Sample No.**	S1	S2	S3-1 (“Amo”)	S4	S5	S6
			S3-2 (“Ann”)			
			S3-3 (“ATO”)			

**Table 2:** Mixed-valence of the vanadium oxide films before and after annealing (S3-1 and S3-2) determined by XPS and AES characterization.

	VO_2_ Ratio	V_2_O_5_ Ratio	O2p: V3d	x (VO_x_)	Mixed-Valence
**S3-1**	38.64%	61.36%	0.81	2.31	4.62
**S3-2**	48.24%	51.76%	0.71	2.26	4.52

**Table 3:** Detailed fitted thickness parameters of the SOM before annealing (S3-1) and after annealing (S3-2). “SO” is the thickness of the surface oxidation layer; “SR” is the thickness of the surface roughness layer; “VO_2_” represents the interior V4+ layer; and “SiO_2_” represents the natural surface oxidation layer on the Si substrate.

Thickness/nm	SO	SR	VO_2_	SiO_2_
**S3-1**	6.0	0	33.7	1.7
**S3-2**	3.1	1.8	36.3	1.7

## Data Availability

Data are contained within the article.

## Abbreviations

The following abbreviations are used in this manuscript:

ALD	Atomic layer deposition
XPS	X-ray photoelectron spectroscopy
AES	Auger electron spectroscopy
SOM	Surface oxidation model
ATO	Air thermal oxidation
XRD	X-ray diffraction
SE	Spectroscopic ellipsometry
GPC	Growth rate per cycle
SEM	Scanning electron microscopy
AFM	Atomic force microscopy

## Appendix A. Ellipsometry Models

Two quantities were measured in the ellipsometric characterization: Ψ and Δ. The tangent of Ψ is the ratio of the magnitudes of p-wave and s-wave reflection coefficients, and Δ is the phase shift induced by the reflection of the phase difference between p-wave and s-wave:

(A1) $$\tan\Psi =\frac{\left|R_{p}\right|}{\left|R_{s}\right|}$$

(A2) $$\Delta =\delta_{1}-\delta_{2}$$

where $\delta_{1}$ and $\delta_{2}$ are the phase difference before and after the reflection, respectively.

Drude and Tauc-Lorentz models were used to describe the optical properties of vanadium oxides. The dielectric constant can be written as follows:

(A3) $$\varepsilon \left(\omega \right)=\varepsilon_{\infty }+\varepsilon_{D}\left(\omega \right)+\sum_{k=1}^{n}\varepsilon_{TL,k}\left(\omega \right)$$

In Equation (A3), the first term $\varepsilon_{\infty }$ is a constant that denotes the contribution of high-frequency electronic transition. $\varepsilon_{D}\left(\omega \right)$ and $\varepsilon_{TL,k}\left(\omega \right)$ stand for the Drude part and the kth Tauc-Lorentz oscillator, which can be further written as

(A4) $$\varepsilon_{D}\left(\omega \right)=-\frac{\omega_{p}^{2}}{\omega^{2}+i\omega \gamma_{p}}$$

(A5a) $${\varepsilon_{TL,k}}^{im}\left(\omega \right)=\left\{\begin{array}{c} \frac{1}{\hbar \omega }\frac{A_{k}E_{0,\;k}C_{k}{\left(\hbar \omega -E_{g}\right)}^{2}}{{\left[{\left(\hbar \omega \right)}^{2}-{E_{0,\;k}}^{2}\right]}^{2}+{C_{k}}^{2}{\left(\hbar \omega \right)}^{2}}\;,\;\;\;\;\;\;\;\;E>E_{g}\\ 0\;,\;\;\;\;\;\;\;\;\;\;\;\;\;\;\;\;\;\;\;\;\;\;\;\;\;\;\;\;\;\;\;\;\;\;\;\;\;\;\;\;\;\;\;\;\;\;\;\;\;\;\;\;\;\;\;\;\;\;\;\;\;\;\;\;\;E\leq E_{g} \end{array}\right.$$

(A5b) $${\varepsilon_{TL,k}}^{re}\left(\omega \right)=\frac{2}{\pi }P\int_{E_{g}}^{\infty }\frac{\xi {\varepsilon_{TL,k}}^{im}\left(\xi \right)}{\xi^{2}-{\left(\hbar \omega \right)}^{2}}d\xi$$

In Equation (A4), $\omega_{p}={\left(Ne^{2}/\varepsilon_{0}m^{*}\right)}^{1/2}$ is the plasma frequency of R-phase VO_2_ (N is the carrier density and $m^{*}$ is the effective mass of electrons), and $\gamma_{p}$ is the broadening parameter which defines the scattering rate of free electrons. Equations (A5a) and (A5b) describe the imaginary and real part of the kth Tauc-Lorentz oscillator, where $A_{k}$, $E_{0,\;k}$, $C_{k}$ are the amplitude, center energy, and broadening parameter, respectively, and $E_{g}$ is the bandgap energy. With ${\varepsilon_{TL,k}}^{im}\left(\omega \right)$, the real part ${\varepsilon_{TL,k}}^{re}\left(\omega \right)$ can be calculated by the Kramers-Kronig integration, as detailed in Equation (A5b), where P is the representation of Cauchy principal value integral.

**Table A1:** The detailed fitting parameters of S3-1, S3-2 and S3-3. For sample S3-2, owing to the existence of a phase transition, both the low-temperature M1 phase and high-temperature R phase can be observed. Dispersion models include Drude and Tauc-Lorentz models.

		$\varepsilon_{0}$	$E_{g}$	${\hbar \omega }_{p}$	${\hbar \gamma }_{p}$	$E_{0,1}$	$A_{1}$	$C_{1}$	$E_{0,2}$	$A_{2}$	$C_{2}$
**S3-1**		2.75	0.47	/	/	1.43	10.22	2.68	2.53	2.86	1.54
**S3-2**	**M1**	3.85	0.50	/	/	0.54	26.15	19.12	3.20	9.09	1.00
	**R**	0.36	0	4.33	0.68	3.17	8.41	1.32	/	/	/
**S3-3**		2.75	0.79	/	/	3.23	14.35	0.98	/	/	/
